# The Relationship Between Clinical Profiles, Glycemic Parameters, and Hypoglycemia in Pediatric Patients with Type 1 Diabetes

**DOI:** 10.3390/jcm15031112

**Published:** 2026-01-30

**Authors:** Andreea Morar-Stan, Luminița Dobrotă, Anișoara Răduțu, Carmen Daniela Domnariu

**Affiliations:** 1 Faculty of Medicine, “Lucian Blaga” University of Sibiu, 550024 Sibiu, Romania; luminita.dobrota@ulbsibiu.ro (L.D.); carmen.domnariu@ulbsibiu.ro (C.D.D.); 2 County Clinical Emergency Hospital of Oradea, 410467 Oradea, Romania

**Keywords:** type 1 diabetes, hypoglycemia, glycemic variability, mean daily glucose, pediatric population

## Abstract

**Background/Objectives**: Our objective was to assess the role of clinical and continuous glucose monitoring (CGM) parameters in predicting the risk of hypoglycemia in pediatric patients with type 1 diabetes. **Methods**: Pediatric patients with type 1 diabetes (*n* = 71) at the Oradea County Clinical Emergency Hospital, Romania, who underwent CGM during their initial visit and were followed for at least 6 months with in-clinic visits every 3 months were enrolled in this study. Age, body mass index, time in range, the mean daily glucose (MDG) concentration, and the coefficient of variation (%CV) were considered as potential predictors of the risk of hypoglycemia, which was defined as the percentage of time spent below two glycemic thresholds of 3.9 and 3.0 mmol/L, corresponding to mild and clinically significant hypoglycemia, respectively. **Results**: Among a total of 142 glycemic profiles, the MDG concentration was significantly lower in those with hypoglycemia compared to those without, whereas %CV was significantly higher (*p* < 0.0001). Regression tree models identified %CV as the dominant variable for both thresholds, whereas classification tree models identified %CV as the dominant variable for clinically significant hypoglycemia and MDG for mild hypoglycemia. In profiles with a %CV of less than 36.15% and an MDG concentration greater than 7.16 mmol/L, the mean percentage of time spent below the 3.9 mmol/L threshold was 4.8%, which is close to that recommended by the American Diabetes Association guidelines. Patients younger than 7 years presented the highest frequency for both mild and clinically significant hypoglycemic episodes. **Conclusions**: Our study supports %CV and the MDG concentration as key factors in predicting hypoglycemia risk. Minimizing the risk of hypoglycemia in pediatric patients requires a %CV of less than 36%.

## 1. Introduction

Type 1 diabetes mellitus (T1DM), known as the most prevalent form of diabetes in the pediatric population, is a chronic autoimmune condition that leads to the progressive destruction of pancreatic β-cells, resulting in an absolute endogenous insulin deficiency and high blood sugar (hyperglycemia) and requiring lifelong insulin therapy for survival [1,2]. Although historically considered a metabolic disorder, current understanding emphasizes T1DM as a complex interplay of genetic susceptibility, immune dysregulation, and environmental triggers [3]. The continuous global rise in T1DM incidence represents a major clinical concern, as it has become one of the leading endocrine disorders of childhood [4,5]. This epidemiological reality requires the adoption of strict glycemic management based on intensive exogenous insulin replacement therapy delivered via multiple daily injections or continuous subcutaneous insulin infusion (CSII), both of which require meticulous titration to approximate physiological insulin secretion patterns. Regarding CSII modalities, technology has evolved to advanced hybrid closed-loop (AHCL) systems, which improve time in range (TIR) without increasing the risk of hypoglycemic events [6,7]. However, the efficacy of treatment is intrinsically linked to glucose monitoring accuracy. The transition from the self-monitoring of blood glucose (SMBG) to continuous glucose monitoring (CGM) is associated with reduced hypoglycemia and substantially improved glycemic control [8,9,10,11].

This shift is particularly essential in the pediatric population, where developmental limitations in identifying and communicating symptoms are prevalent. The integration of predictive alert systems within CGM devices serves as a safety net, identifying occult hypoglycemic excursions that often elude both the patient and the caregiver [12]. Although management strategies for type 1 diabetes have advanced considerably, hypoglycemia remains the most common and most dangerous acute complication with increased risks of acute morbidity and long-term mortality [13,14]. In clinical practice, the definition of hypoglycemia has evolved beyond a subjective symptomatic definition toward a classification based on three distinct levels: level 1, glucose ≤ 70 mg/dL, representing a glycemic alert value that requires clinical attention; level 2 (glucose ≤ 54 mg/dL), indicating clinically significant hypoglycemia; and level 3, characterized by severe cognitive impairment requiring external assistance for recovery [15,16].

Numerous factors have been implicated in the pathogenesis of hypoglycemia, most notably the mismatch between administered insulin and nutritional intake. Hypoglycemic episodes may result from a combination of iatrogenic and behavioral determinants, including a suboptimal understanding of insulin type and action, accidental delivery, and nutritional deficits such as reduced food intake or fasting, and in situations where glucose utilization is increased (during exercise) or endogenous glucose production is decreased (after alcohol intake) [17].

Children with T1DM frequently experience the consequences of recurrent hypoglycemia, including disrupted sleep, poor academic performance, and social withdrawal. These stressors contribute to the emergence of fear of hypoglycemia (FoH) as well as increased anxiety and depression [18,19]. In this context, the ambulatory glucose profile (AGP), which is widely recognized as the gold standard for the visualization and clinical interpretation of CGM metrics, functions as a vital medical instrument. The implementation of AGP reports in pediatric clinical practice has been demonstrated to be a feasible option, thereby allowing the continuous assessment of glycemic status and, implicitly, the identification of hypoglycemic episodes that may otherwise remain undetected during routine clinical care [20]. According to the international consensus on the use of CGM, among the parameters quantified using AGP, the coefficient of variation (%CV) is considered a key indicator distinguishing stable from unstable diabetes, with superior sensitivity for detecting hypoglycemic excursions. Beyond a %CV of 36%, the frequency of hypoglycemia is significantly increased, particularly in insulin-treated patients [21,22]. In addition to %CV, the mean daily glucose (MDG) concentration appears to be another major explanatory factor contributing to hypoglycemia risk. However, only a few studies have investigated the appropriateness of these thresholds in pediatric patients, with this study being the first one conducted in Romania.

Therefore, this study aims to fill the gap through a retrospective observational analysis by evaluating how clinical and CGM parameters correlate with hypoglycemia in a local context, thus providing a baseline for national pediatric care.

## 2. Materials and Methods

We conducted a retrospective observational study using data from patients with T1DM admitted to the Department of Diabetology and Nutrition Diseases, Pediatric I of the Oradea County Clinical Emergency Hospital. The main objective of this study was to analyze the role of clinical and CGM parameters in determining the time spent below the hypoglycemia thresholds of 3.9 mmol/L and 3.0 mmol/L.

The present study was approved by the Ethics Committee of the Oradea County Emergency Clinical Hospital (No. 19384/26 June 2025) and the “Lucian Blaga” University of Sibiu (No.57/12 November 2025). The present study was performed in accordance with the ethical principles outlined in the Declaration of Helsinki.

Inclusion criteria were a type 1 diabetes diagnosis according to the American Diabetes Association classification, ages between 2 and 18 years, users of real-time CGM (rtCGM) systems, a well-defined basal-bolus insulin regimen in the last 3 months, parental consent, and willingness and ability to adhere to the study protocol, along with an information system that met the requirements for uploading study data [23]. Exclusion criteria for analysis were other forms of diabetes, absence of data for any of the studied variables, unexpected interruption of glucose monitoring, corticosteroid therapy in the last 3 months, comorbidities (celiac disease and autoimmune thyroiditis), and pregnancy.

Therapeutic management was divided between multiple day injections (MDIs) and continuous subcutaneous insulin infusion (CSII). The CSII group exclusively utilized Medtronic MiniMed^TM^720G and 740G systems (Medtronic, Heerlen, The Netherlands). The use of these specific models is attributed to the phased rollout and reimbursement protocols for AHCL, which dictated technology access during the study period. To ensure compatibility with the MDI group regarding insulin dosing decisions, all pumps systems were operated in manual mode, requiring user-initiated boluses and manual basal rate adjustments.

Glycemic control and hypoglycemia risk were assessed using the ambulatory glucose profile (AGP) report.

Patients were asked to wear an rtCGM device (Guardian^TM^4 sensor, Medtronic, Heerlen, The Netherlands) for the CSII group, with glycemic data subsequently retrieved and analyzed using CareLink™ Personal software (v.5.0, Medtronic), and a Dexcom one + sensor (Dexcom Inc., San Diego, CA, USA) with data retrieved through Dexcom Clarity^®^ software (v3.50.3, Dexcom Inc., San Diego, CA, USA) for the MDI group during their initial visit and were followed for at least 6 months, with in-clinic visits every 3 months. RtCGM data were uploaded at every visit. For each patient, two AGPs were obtained over a 6-month interval. Consequently, the final analysis sample included 142 profiles. Regarding data from subjects included in the analyses, at least 90 days of consecutive CGM wear with a sensor wear time ≥70% was required based on previous guidelines [24,25,26]. Data analysis followed international consensus guidelines for CGM metrics, focusing on glucose management indicators (GMIs), time in range (TIR), the coefficient of variation (%CV), and mean daily glucose (MDG).

All analyses were run using R (version 4.5.2). The Wilcoxon rank sum test with continuity correction was conducted using the Wilcox test function to compare means between two groups.

Univariate linear models were used to explore the existence of an association between explanatory variables, including age, body mass index (BMI), MDG, %CV, TIR, and insulin dose, and response variables, including percentage of time spent below glucose thresholds of 3.0 and 3.9 mmol/L. The arcsin square root transformation was applied to the response variable. Mixed models with a random intercept for participants were chosen, as two glycemic profiles were available per participant, and the models were run using the lmer command from the lme4 package, version 1.1.38, e.g., lmer(arcsin(sqrt(percentage of time spent below threshold/max(percentage of time spent below threshold))~age + (1|participant), data = dataset)). The ANOVA function from the car package version 3.1.3 was used to perform Type II Wald chi-square tests to assess the significance of each explanatory variable. Multivariate linear mixed models were also fitted, including all variables that were significant in the univariate analysis; all variables remained significant in the models.

Recursive partitioning and regression trees were fitted using the rpart function in the rpart package version 4.1.24 and plotted using the fancy RpartPlot function from the rattle package version 5.1.5. These trees are read from top to bottom and generate predictions following a series of if/then questions based on the considered features. All variables that were significant in the univariate linear mixed-model analysis were included as explanatory variables. The response variables included the percentage of time spent below glucose thresholds of 3.0 (clinically significant hypoglycemia) and 3.9 mmol/L (mild hypoglycemia) (regression) as well as any time versus no time spent below each glucose threshold (classification). For each tree, a complexity parameter table was plotted using the plotcp function, and the tree depth corresponding to the first value on the left of the plot that lies below the horizontal line was chosen.

Beta regression was used to model the relationship of the percentage of time spent below the glucose thresholds with MDG and %CV separately. This type of regression is specifically designed to analyze continuous variables that are bounded between 0 and 1 and avoids unrealistic predictions outside this range. Percentages were divided by 100, and 0.001 was added to all values because the beta distribution excludes 0. This constant was then subtracted from the predictions before multiplying by 100. These models were fitted using the betareg package version 3.2.4. For comparison and following a previous study [27], a linear model was also fitted on the log-transformed data after applying the same transformations, as the natural logarithm is undefined at 0.

To examine the relationship between age categories (<7, 7–13, and >13 years) and the risk of mild and clinically significant hypoglycemic events, we created two-way contingency tables and conducted the chi-square test of independence, testing for evidence against the null hypothesis of no relationship (independence).

## 3. Results

Our study had a total of 71 participants, each with two glycemic profile measurements (142 glycemic profiles). Of these, 32 were receiving continuous subcutaneous insulin infusion (CSII), and 39 were on multiple daily injections (MDIs). Ages ranged from 3 to 18 years, with a median of 13 (interquartile range (IQR): 9–15). In total, 36 (51%) were males. BMI ranged from 14.10 to 31.10, with a median of 20.00 (IQR 18.30–22.05). Regarding diabetes duration, 6 (8%) patients had diabetes for less than 1 year, 31 (44%) had diabetes between 1 and 5 years, and 34 (48%) had it for more than 5 years. The mean HbA1C was 7.9, with a standard deviation (SD) of 1.9 and a range of 5.3–15.1 (*n* = 71). The mean MDG was 8.1, with an SD of 1.2 and a range of 5.9–13.4 (*n* = 142). The mean %CV was 39.1%, with an SD of 6.1% and a range of 23.8–61.1% (*n* = 142). The mean %TIR was 60.2%, with an SD of 16.8% and a range of 20–100%. The mean daily insulin dose was 0.89, with an SD of 0.36 and a range of 0.16–2.05 (*n* = 71). In total, 36 out of 142 (25%) patients had a TIR > 70%. The median GMI was 7.1 (IQR: 6.7–7.5).

Glycemic profiles were categorized according to whether any time was spent below glucose thresholds versus no time. Two glucose thresholds were considered, namely 3.0 mmol/L, corresponding to clinically significant hypoglycemia, and 3.9 mmol/L, corresponding to mild hypoglycemia. The number of glycemic profiles with any time spent below 3.0 mmol/L was less than the number with any time spent below 3.9 mmol/L (Table 1). MDG was significantly lower (Wilcoxon test *p* < 0.0001) in those with both clinically significant and mild hypoglycemia compared to those without, where as %CV was significantly higher (*p* < 0.0001). No difference in %TIR was noted between those with and without clinically significant hypoglycemia; however, %TIR was significantly higher in those with mild hypoglycemia compared to those without (Wilcoxon test: *p* = 0.01).

No differences in mean age, BMI, or insulin dose were noted between those with and without hypoglycemia. There was no evidence of a difference in mean GMI for clinically significant hypoglycemia; however, a difference was noted for mild hypoglycemia. All glycemic profiles with no percentage of time spent below 3.0 mmol/L had a %CV < 36.1%, whereas all those with any time spent below the threshold had a %CV > 36.0% (Table 1). These results indicating that, at least in our dataset, a %CV threshold of 36.0% nearly perfectly distinguishes those with clinically significant hypoglycemia from those without. We note, however, that this distinction was less clear for mild hypoglycemia with %CV overlapping between 29.1% and 35.4% for participants with and without events. Similarly, an overlap in MDG was observed for both clinically significant and mild hypoglycemia.

Table 1 presents glycemic profiles categorized based on any time versus no time, whereas Table 2 presents these data based on the categorization of time below ranges (TBRs) > 1% (3.0 mmol/L) and >4% (3.9 mmol/L), together with the means and standard deviations for age, BMI, MDG, %CV, %TIR, and insulin dose.

Considering one explanatory variable at a time, linear mixed models found statistically significant associations for the explanatory variables MDG and %CV with the response variables percentage of time spent below glucose thresholds of 3.0 and 3.9 mmol/L (Type II Wald chi-square test, *p* < 0.0001 for all but MDG with clinically significant hypoglycemia, which had a *p* = 0.0005). No evidence of association was found for age, BMI, or insulin dose (*p* > 0.2). Regarding %TIR, there was no evidence of an association with the percentage of time spent below 3.9 mmol/L (*p* = 0.8). However, a statistically significant association with the percentage of time spent below 3.0 mmol/L was observed (*p* = 0.02). Multivariate linear mixed-model results showed that the percentage of time spent below the thresholds increased significantly with %CV but decreased significantly with MDG (*p* < 0.001) as well as with TIR, although only marginally (Table 3).

**Figure 1:**
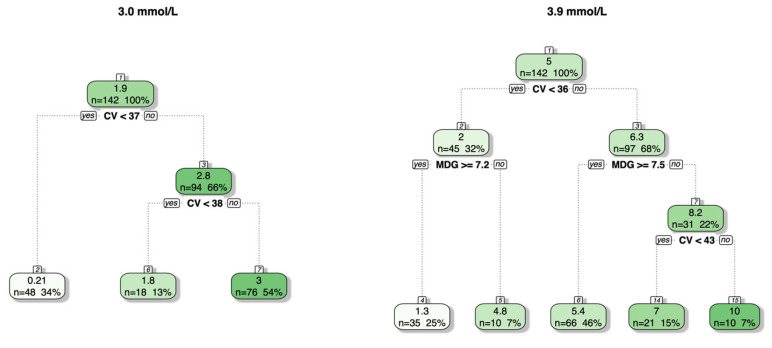
Regression trees based on %CV and MDG for predicting the percentage of time spent below the two glucose thresholds of 3.0 mmol/L (clinically significant hypoglycemia) and 3.9 mmol/L (mild hypoglycemia). At each internal node, the label (e.g., CV < 37) indicates the condition for the left-hand branch, with the right-hand branch corresponding to the inverse condition (CV ≥ 37). Leaf nodes show the mean predicted percentage of time spent below the two glucose thresholds in that specific subgroup. The numbers at the top of each box represent the node number, with jumps being present when the tuning of the tree resulted in the removal of branches. The gradient reflects the mean of the response variable for the specific split, with a lower percentage of time represented by lighter shades and a higher percentage of time by darker shades.

Recursive partitioning and regression trees that included both MDG and %CV were fitted for percentage of time spent below both glucose thresholds (Figure 1). For clinically significant hypoglycemia, trees that considered splitting based on %TIR were also generated; however, no changes in the trees were observed.

For both glucose thresholds, a %CV of approximately 36% separated the glycemic profiles with the lowest percentage of time spent below the threshold. Interestingly, in clinical practice, <1% of the time below the threshold is often considered acceptable for clinically significant hypoglycemia, and <4% is often considered acceptable for mild hypoglycemia. These cutoffs are also reflected in the splits identified by the trees (mean percentage of time at the leaves: 0.2% and 1.8% for clinically significant and 4.8% and 5.4% for mild hypoglycemia). For clinically significant hypoglycemia, a further split on %CV identifies cases with an even higher percentage of time, although even the most extreme percentages of time remain relatively small in our dataset. For mild hypoglycemia, the second split is based on MDG, with the highest percentage of time spent below the 3.9 mmol/L threshold corresponding to %CV values greater than 43.2%.

**Figure 2:**
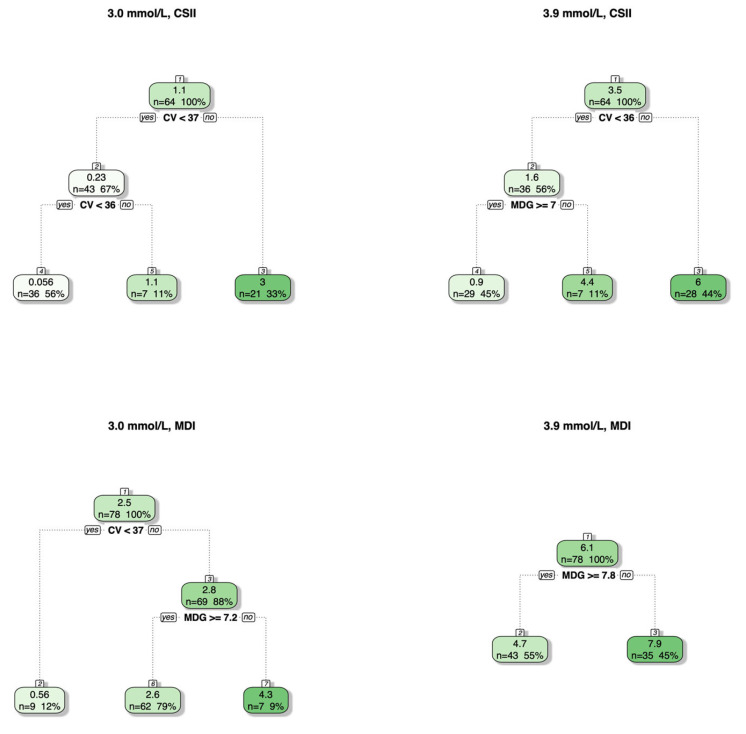
Regression trees for predicting the percentage of time spent below the two glucose thresholds of 3.0 mmol/L (clinically significant hypoglycemia) and 3.9 mmol/L (mild hypoglycemia) based on %CV and MDG and stratified by patients who received CSII versus MDI. The numbers at the top of each box represent the node number, with jumps being present when the tuning of the tree resulted in the removal of branches. The gradient reflects the mean of the response variable for the specific split, with a lower percentage of time represented by lighter shades and a higher percentage of time by darker shades.

In total, 23 out of 64 (36%) glycemic profiles from patients receiving CSII had a TBR > 1% (3.0 mmol/L threshold), compared to only 71 out of 78 (91%) from patients receiving MDI. In total, 25 out of 64 (39%) glycemic profiles from patients receiving CSII had a TBR > 4% (3.9 mmol/L threshold), compared to only 56 out of 78 (72%) from patients receiving MDI. Considering regression trees for glycemic profiles stratified by CSII versus MDI (Figure 2), these trees were very similar to those constructed using all glycemic profiles. The exception was the MDI group at the 3.9 mmol/L threshold, where the model produced only one split on MDG ≥ 7.8. Notably, this is the only tree in which the leftmost leaf has a mean percentage of time spent below the threshold >1%, making it unable to further split the profiles with >1% of time below the threshold.

**Figure 3:**
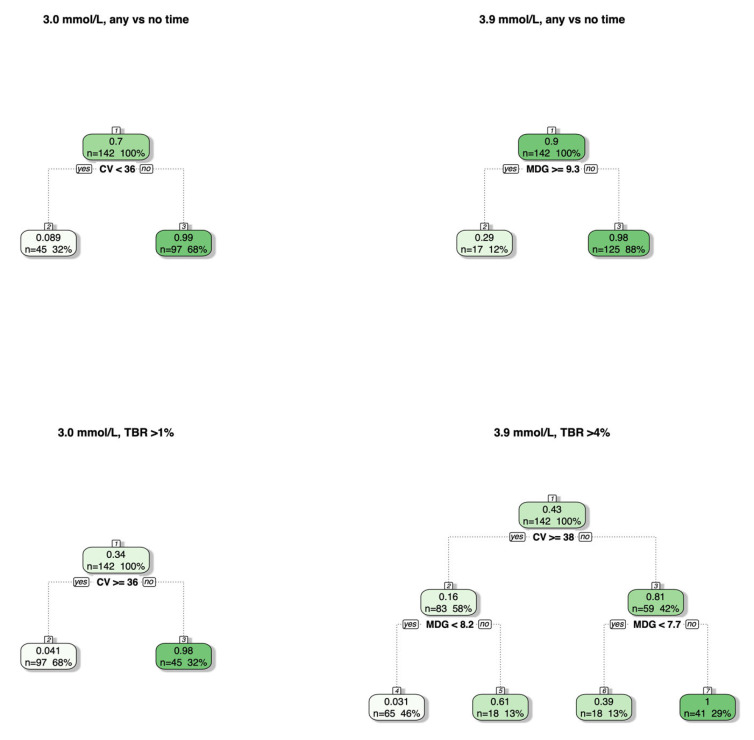
Classification trees for predicting any percentage of time versus no time (first row) spent below the two glucose thresholds of 3.0 mmol/L (clinically significant hypoglycemia) and 3.9 mmol/L (mild hypoglycemia) along with TBRs > 1% and >4% (second row) while using CV and MDG as the input features. The numbers at the top of each box represent the node number, with jumps being present when the tuning of the tree resulted in the removal of branches. The gradient reflects the mean of the response variable for the specific split, with lighter shades illustrating a small percentage of glycemic profiles with any percentage of time below the threshold, and darker shades a high percentage of glycemic profiles with any percentage of time below the threshold.

When considering classification trees for any percentage of time versus no time spent below the glucose thresholds, trees with a depth of one are chosen for both thresholds, with a split on %CV (36%) for clinically significant hypoglycemia and on MDG (9.3 mmol/L) for mild hypoglycemia (Figure 3). Specifically, all patients with a %CV above 36% are predicted to have experienced clinically significant hypoglycemia, whereas those with a %CV below 36% were predicted to have spent no time below 3.0 mmol/L. All patients with an MDG below 9.3 mmol/L were predicted to have experienced mild hypoglycemia, whereas those with an MDG above 9.3 mmol/L were predicted to have spent no time below 3.9 mmol/L. When considering classification trees for TBR > 1% (3.0 mmol/L threshold) and TBR > 4% (3.9 mmol/L), there were no changes for clinically significant hypoglycemia. However, for mild hypoglycemia, the first split was on %CV ≥ 38%, followed by additional splits on MDG < 7.7 and MDG < 8.2 (Figure 3).

**Figure 4:**
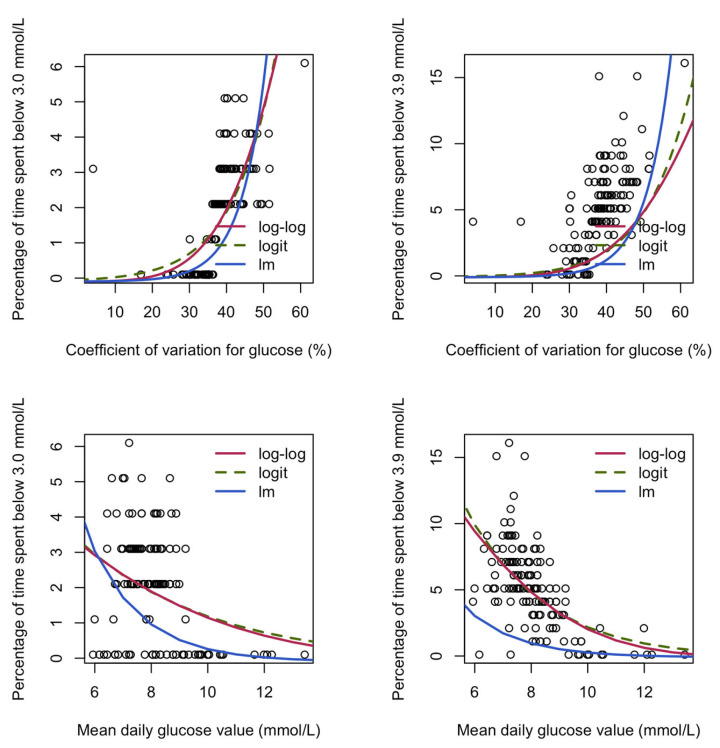
Predictions for the percentage of time spent below the two glucose thresholds of 3.0 mmol/L and 3.9 mmol/L from regression models while using the coefficient of variation for glucose (%) and the mean daily glucose value (mmol/L) as explanatory variables. Models presented include the beta regression models using log–log and logit links, as well as linear regression (lm) on log-transformed data. Each dot corresponds to a glycemic profile. Note: For all models, percentages were divided by 100 to scale them to [0, 1], and 0.001 was added to all response variables because the beta distribution does not include 0 and 1, and the natural logarithm of 0 is not defined. The constant 0.001 was subtracted from the predictions.

Considering the relationship between %CV and MDG and the percentage of time spent below glucose thresholds, the percentage of time increased as %CV increased for both thresholds, and it decreased as MDG increased (Figure 4), regardless of the regression model used.

Considering the relationship between age (<7, 7–13, and >13 years) and any time spent below the glucose thresholds of 3.0 mmol/L and 3.9 mmol/L, there was weak evidence (*p* = 0.1 and *p* = 0.07) for a relationship between the two categorical variables (Table 2). The percentage of both clinically significant and mild hypoglycemia was highest in the <7-year age category, with similar percentages for 7–13 years and >13 years for clinically significant hypoglycemia. However, a lower percentage of mild hypoglycemia was noted in children 7–13 years compared to those >13 years (Table 4).

## 4. Discussion

The results obtained in the present study highlight the key parameters predictive of hypoglycemic episodes among children and adolescents with T1DM. MDG was significantly lower (Wilcoxon test: *p* < 0.0001) in those with both clinically significant and mild hypoglycemia compared to those without, whereas %CV was significantly higher (*p* < 0.0001). This finding aligns with the recent analysis of the ISCHIA study by Murata et al. (2025) [23], who demonstrated that the risk of hypoglycemia is not determined by a single metric but by the interaction between average glucose and variability. Their study demonstrated that a lower MDG level reduces the “safety margin”, where as a high %CV provides the oscillations that trigger hypoglycemic events [23].

The percentage of time spent below glucose thresholds increased as %CV increased for both thresholds, and it decreased as MDG increased. In addition, %CV and MDG were significantly associated with the presence of hypoglycemia in adults in a study by Monnier et al., where their regression tree models identified %CV as the dominant variable for the 3.0 mmol/L threshold [27]. MDG was identified as the dominant variable for the 3.9 mmol/L threshold; however, the percentage of time spent below the thresholds in their study was more extreme compared with our study, with our highest mean percentage of time spent below 3.9 mmol/L being 10.5% versus 37% in their regression tree.

Consistent with international consensus, our results confirm that the risk of clinically significant hypoglycemia was minimal when %CV values were below 36%. Our findings regarding the exponential increase in hypoglycemia risk as %CV rises are consistent with the work by Piona et al. (2021) [28]. They demonstrated that in children and adolescents, high glycemic variability is fundamentally linked to worse CGM metrics, particularly an increase in the time below range (TBR) [28]. Consistent with the evidence provided by their study, our results further suggest that stabilizing %CV should be the primary focus in routine pediatric visit. One of the strengths of our analysis was the beta regression models, which are specifically designed to model continuous data bounded between 0 and 1; however, we also included an exponential fit for comparison with this study.

It was observed that in profiles with a %CV below 36.15% and MDG levels above 7.16 mmol/L, the mean time spent below the 3.9 mmol/L threshold was 4.8%, a value close to the 4% value recommended by the American Diabetes Association guidelines [29]. This conclusion suggests that pediatric patients with lower glucose variability may achieve better glycemic control with a lower risk of hypoglycemia, consistent with findings reported by Urakami et al. [30]. According to our regression tree model results, an MDG concentration of 9.3 mmol/L was predictive of eliminating any time spent below the 3.9 mmol/L threshold. This MDG value exceeds the upper limit of euglycemia—7.8 mmol/L [31]—reinforcing the results from the specialized literature that shows that fear of hypoglycemia, often manifested through caregiver-driven overcorrection of glucose levels, may contribute to suboptimal glycemic control, particularly in the pediatric population [32,33,34]. In addition, the present analysis revealed that patients utilizing integrated insulin pump systems achieved significantly lower glycemic variability (%CV) compared to those on MDI. Specifically, our results align with the population-based study by Karges et al., demonstrating that CGM and pump therapy are associated with a reduced risk of severe hypoglycemia in children and adolescents [35].

Until recently, only a small proportion of individuals with T1DM were able to simultaneously achieve such targets—low %CV and a near-normal MDG concentration—using conventional insulin therapy, whether through multiple daily injections or CSII using an insulin pump. Several factors have been identified as key contributors to hypoglycemia risk in the pediatric population, most notably age and dietary patterns, which exhibit a complex, reciprocal influence on one another. The literature emphasizes that the accuracy of carbohydrate counting by both children and parents is critical for postprandial glycemic stability [36]. Furthermore, meals high in fats and proteins can delay glucose absorption, potentially leading to early hypoglycemia [37]. However, in younger children, the lack of predictability in food intake appears to be one of the most challenging factors regarding hypoglycemic events [38]. Behavioral factors also play a significant role; for instance, dietary restrictions or fasting, particularly among adolescents, can trigger hypoglycemic episodes, which are often followed by compensatory over consumption [39]. Clinically significant hypoglycemia has been associated with ages under 5 years, with the likelihood of developing hypoglycemia being approximately six times higher in this age group compared to children over 10 years [40]. In the studied cohort, patients younger than 7 years demonstrated the highest frequency for both mild and clinically significant hypoglycemic episodes. This observation is of particular importance given that recurrent and early exposure to hypoglycemia predisposes patients to maladaptive physiological responses.

Because the iatrogenic development of impaired hypoglycemia awareness and autonomic failure are considered mediators of severe hypoglycemia, medical efforts to manage both mild and clinically significant hypoglycemic episodes should be prioritized, especially in pediatric patients, whose age at disease onset has shown concerning trends in recent years [41,42,43,44]. Furthermore, evidence from large-scale clinical trials, real-world studies, and meta-research supports the association between hypoglycemia and adverse cardiovascular outcomes in patients with diabetes, reinforcing the long-standing principle attributed to Erasmus of Rotterdam, “prevention is better than cure,” and underscoring the importance of preventing this complication from early childhood [45,46,47]. Integrating stable clinical factors (such as age at evaluation) with dynamic indicators of glycemic control (%CV, MDG, TIR, GMI, and TBR) enables predictive and proactive stratification of pediatric patients at increased risk for recurrent and clinically significant hypoglycemia.

While the current analysis establishes the coefficient of variation (%CV) as a robust and evidence-based predictor of glycemic stability, we acknowledge that expanding the metric set with advanced glycemic indices such as the low blood glucose index (LBGI) and the high blood glucose index (HBGI) could further enhance clinical granularity. Future prospective studies on the pediatric population should integrate these indices along with TIR, %CV, mean daily glucose, and TBR. Although TBR provides a comprehensive overview of hypoglycemia, analyzing the circadian distribution, frequency, and duration of hypoglycemic episodes represents a key future research direction to better understand time-specific risks in the pediatric population. Future research should explore how glycemic variability and mean daily glucose contribute to the fear of hypoglycemia experienced by both patients and caregivers. Accordingly, the development and rigorous evaluation of psychotherapeutic interventions tailored to the pediatric population are essential.

Several limitations must be acknowledged. This study has an observational design and a relatively short monitoring period for AGPs. Although CGM remains a transformative tool in pediatric diabetes care, its integration must account for the interstitial-to-blood glucose delay and the potentially lower sensor precision during hypoglycemia (MARD). Our findings underscore the need for clinical oversight and, as recommended by the ADA, confirmation using a blood glucose meter (BGM) when CGM readings do not correlate with clinical symptoms or when rapid glucose fluctuations occur [29]. This study’s findings may be influenced by the indispensable use of hypoglycemia alerts due to the vulnerability of the pediatric group age, combined with caregiver anxiety, which may lead to aggressive intervention. A further limitation of this study was the inability to standardize physical activity, dietary patterns, and bolus correction due to the age-related heterogeneity of the cohort and the participants’ substantial reliance on caregivers for daily management.

## 5. Conclusions

In conclusion, the findings of our study provide a clear answer to the proposed research question by demonstrating that glycemic variability (assessed using the coefficient of variation), MDG levels, and patient age at evaluation are key factors in predicting and preventing hypoglycemia risk. Based on these results, achieving and maintaining a %CV of less than 36% may serve as a primary target within strategies aimed at reducing the incidence of hypoglycemic episodes in children and adolescents with type 1 diabetes.

## Figures and Tables

**Table 1:** Glycemic profiles categorized by any time spent below two glucose thresholds (3.0 and 3.9 mmol/L)versus no time, with the corresponding mean, standard deviation (SD), and minimum and maximum values for age, BMI, MDG, %CV, TIR, insulin dose, and GMI.

	Glucose Thresholds			
	Any Percentage of Time Spent Below 3.0 mmol/L (Clinically Significant Hypoglycemia)		Any Percentage of Time Spent Below 3.9 mmol/L (Mild Hypoglycemia)	
	No	Yes	No	Yes
Glycemic profiles (*n*)	42	100	14	128
	Mean (SD), min–max			
Age (years)	13 (3),	12 (4),	12 (2),	12 (4),
	4–18	3–18	8–15	3–18
BMI (kg/m^2^)	20.8 (3.1),	20.4 (3.6),	19.6 (3.3),	20.7 (3.4),
	16.5–30.0	14.1–31.1	16.6–30.0	14.1–31.1
MDG (mmol/L)	8.9 (1.8),	7.8 (0.6),	10.2 (1.9),	7.9 (0.9),
	5.9–13.4	6.0–9.2	6.2–13.4	5.9–12.0
%CV	32.3 (3.2),	41.8 (4.8),	31.1 (4.1),	40.1 (5.6),
	23.8–36.1	36.0–61.1	23.8–35.4	29.1–61.1
%TIR	64.2 (21.2),	58.5 (14.3),	50.1 (22.4),	61.3 (15.8),
	27.0–100.0	20.0–95.0	27.0–100.0	20.0–99.0
GMI	7 (1),	7 (1),	8 (1),	7 (1),
	6–9	6–8	6–9	6–8
Insulin dose (units/kg/day)	0.83 (0.4),	0.92 (0.33),	0.84 (0.41),	0.9 (0.35),
	0.16–2.05	0.16–2.05	0.28–1.81	0.16–2.05

**Table 2:** Glycemic profiles categorized based on time spent below two glucose thresholds (3.0 and 3.9 mmol/L), with TBRs > 1% and >4%, along with the mean and standard deviation (SD) for age, BMI, MDG, %CV, TIR, and insulin dose.

	Glucose Thresholds			
	Percentage of Time Spent Below 3.0 mmol/L >1%		Percentage of Time Spent Below 3.9 mmol/L >4%	
	No	Yes	No	Yes
Glycemic profiles (*n*)	48	94	61	81
	Mean (SD)			
Age (years)	13 (3)	11 (4)	12 (4)	12 (4)
BMI (kg/m^2^)	20.7 (2.9)	20.5 (3.7)	20.6 (3.3)	20.7 (3.6)
MDG (mmol/L)	8.8 (1.8)	7.8 (0.6)	8.9 (1.4)	7.5 (0.6)
%CV	33 (3)	42 (5)	35 (5)	42 (5)
%TIR	65.1 (20.8)	57.7 (13.8)	60.7 (17.3)	59.8 (16.5)
Insulin dose (units/kg/day)	0.86 (0.41)	0.91 (0.32)	0.88 (0.41)	0.91 (0.31)

**Table 3:** Summary of multivariate linear mixed models.

	3.0 mmol/L		
Predictors	Estimates	CI	p
(Intercept)	1.37	0.74–2.00	<0.001
%CV	0.02	0.02–0.03	<0.001
MDG	−0.16	−0.20–−0.12	<0.001
TIR	−0.01	−0.01–−0.01	<0.001
Random Effects			
σ2	0.05		
τ00 patient	0.00		
ICC	0.08		
N patient	71		
Observations	142		
Marginal R^2^/Conditional R^2^	0.605/0.636		
	**3.9 mmol/L**		
**Predictors**	**Estimates**	**CI**	***p***
(Intercept)	1.44	1.00–1.87	<0.001
%CV	0.01	0.01–0.02	<0.001
MDG	−0.15	−0.18–−0.12	<0.001
TIR	−0.00	−0.01–−0.00	0.001
Random Effects			
σ2	0.01		
τ00 patient	0.01		
ICC	0.49		
N patient	71		
Observations	142		
Marginal R^2^/Conditional R^2^	0.646/0.820		

**Table 4:** Contingency table for age (<7, 7–13, and >13 years) and any time spent below the glucose thresholds of 3.0 mmol/L and 3.9 mmol/L together with Pearson’s chi-squared test to assess the relationship between these two categorical variables.

	Any Time Spent Below the Glucose Threshold			
	3.0 mmol/L		3.9 mmol/L	
Age	No	Yes	No	Yes
<7 years	1 (7%)	13 (93%)	0 (0%)	14 (100%)
7–13 years	22 (31%)	50 (69%)	11 (15%)	61 (85%)
>13 years	19 (34%)	37 (66%)	3 (5%)	53 (95%)
Pearson’s chi-squared test	X^2^ = 3.92, df = 2, *p*-value = 0.1	X^2^ = 5.19, df = 2, *p*-value = 0.07	X^2^ = 3.92, df = 2, *p*-value = 0.1	X^2^ = 5.19, df = 2, *p*-value = 0.07

## Data Availability

The datasets presented in this article are not readily available because they belong to the Department of Diabetology and Nutrition Diseases of the Oradea County Clinical Emergency Hospital. Requests for access the datasets should be directed to the corresponding author.

## Abbreviations

The following abbreviations are used in this manuscript:

CGM	continuous glucose monitoring
CV	coefficient of variation
MDG	mean daily glucose
T1DM	type 1 diabetes mellitus
CSII	continuous subcutaneous insulin infusion
MDIs	multiple daily injections
AGP	ambulatory glucose profile
rtCGM	real-time continuous glucose monitoring
TIR	time in range
BMI	body mass index
IQR	interquartile range
SD	standard deviation
TBR	time below range
